# Dual-Functional Self-Assembled Nanoparticles for Synergistic Photodynamic Therapy and Antimetastatic Treatment of Colorectal Cancer

**DOI:** 10.3390/pharmaceutics18080948

**Published:** 2026-07-31

**Authors:** Yixuan Li, Haokun Zhang, Tinghai Xu, Ruifeng Jiang, Yubin Zhu, Dong Wang, Peng Xu

**Affiliations:** 1 Key Laboratory of Immune Microenvironment and Inflammatory Disease Research in Universities of Shandong Province, School of Basic Medical Sciences, Shandong Second Medical University, Weifang 261053, China; liyixuan030207@163.com (Y.L.); haokunz2026@163.com (H.Z.); guanfenghai1@163.com (T.X.); RuifengJ2026@163.com (R.J.); zhuerding@zhuerding.top (Y.Z.); 2 School of Clinical Medicine, Shandong Second Medical University, Weifang 261053, China; 3 College of Biological Science and Engineering, Fuzhou University, Fuzhou 350108, China

**Keywords:** colorectal cancer, uPA inhibitor, self-assembled nanoparticles, photodynamic therapy, anti-metastatic therapy

## Abstract

**Background:** Colorectal cancer (CRC) is a major clinical challenge due to high metastasis and therapy resistance. Photodynamic therapy (PDT) offers precise tumor ablation but lacks sustained anti-metastatic activity. Peptidic urokinase-type plasminogen activator (uPA) inhibitors suppress metastasis but suffer from short half-life and poor tumor retention. This study aimed to develop a dual-functional self-assembled nanoplatform integrating PDT and selective uPA inhibition for synergistic CRC treatment. **Methods**: We designed and synthesized a conjugate by linking pyropheophorbide-a (PPA) with uPA-targeted cyclic peptide IG2, which self-assembled into nanoparticles (PINPs). Physicochemical properties, reactive oxygen species (ROS) generation, and uPA inhibitory activity were characterized. In vitro studies included cellular uptake, cytotoxicity, and invasion assays. In vivo therapeutic efficacy was evaluated in subcutaneous CT26 tumor models and lung metastasis models, with biosafety assessed by body weight monitoring. **Results**: PINPs exhibited uniform spherical nanostructure, prolonged blood circulation, and enhanced tumor accumulation via the enhanced permeability and retention (EPR) effect. Under 680 nm irradiation, PINPs generated robust ROS and induced tumor cell apoptosis. PINPs potently inhibited uPA activity and suppressed tumor cell invasion. In vivo, PINPs plus PDT achieved significant tumor growth inhibition (73.6%) and strong anti-metastatic efficacy (60.7%), superior to free IG2. No obvious systemic toxicity was observed. **Conclusions**: The dual-functional PINPs achieve short-term acute tumor ablation via PDT and sustained anti-metastatic potential via uPA inhibition within the tested observation windows, with favorable biosafety. This carrier-free self-assembly strategy provides proof-of-concept validation and a generalizable design paradigm for developing synergistic anti-metastatic nanotherapeutics against metastatic CRC.

## 1. Introduction

Colorectal cancer (CRC) remains one of the most aggressive and life-threatening malignancies worldwide, with high incidence, strong invasiveness, high metastatic potential, and frequent postoperative recurrence [1,2]. Despite continuous improvements in surgical resection, chemotherapy, and targeted therapy, the clinical prognosis of CRC patients remains far from satisfactory [3], mainly due to the lack of effective strategies that can simultaneously eradicate primary tumors and suppress distant metastasis. The inherent limitations of conventional therapies have led to poor long-term survival and underscored the urgent clinical demand for safer, more efficient, and multifunctional therapeutic approaches [4,5]. Recent high-impact clinical studies have demonstrated that prospective, patient-driven clinical research can uncover deep biological mechanisms of tumor progression, therapeutic response, and metastatic dissemination that cannot be fully captured by preclinical or retrospective models [6,7]. These clinical advances highlight the critical value of developing mechanism-driven, multifunctional therapeutic strategies with translational potential, which provides the clinical context and motivation for the present preclinical work.

Photodynamic therapy (PDT) has emerged as a promising minimally invasive strategy for CRC treatment [8,9,10]. Given the anatomical features of the intestinal tract that facilitate light delivery and irradiation, PDT enables highly spatiotemporally controlled local tumor ablation. Upon activation by light at a specific wavelength, photosensitizers generate cytotoxic reactive oxygen species (ROS) to selectively eliminate tumor cells with minimal damage to adjacent normal tissues [11]. In this study, pyropheophorbide-a (PPA), a representative second-generation porphyrin photosensitizer, was selected as the photodynamic module. It possesses a high molar extinction coefficient at 680 nm, robust ROS generation capacity and negligible dark cytotoxicity, which are highly suitable for photodynamic ablation of gastrointestinal tumors [12]. These distinctive advantages make PDT a highly promising candidate for interventional and precision therapy of CRC.

Nevertheless, PDT monotherapy suffers from several critical drawbacks that limit its clinical efficacy [13,14]. First, conventional photosensitizers lack active tumor-targeting capacity, resulting in insufficient tumor accumulation and compromised therapeutic potency. Second, PDT is only effective during light irradiation and fails to provide sustained antitumor activity after light termination, making it difficult to control residual and circulating tumor cells over the long term. Third, PDT is effective mainly against solid primary tumors but is nearly ineffective against invasive or already metastasized cancer cells, which are the leading cause of CRC-related mortality. These intrinsic shortcomings impede the achievement of satisfactory outcomes with PDT alone in advanced and metastatic CRC.

Targeted therapy selectively blocks key signaling pathways in tumor progression by recognizing tumor-associated antigens (TAAs) [15,16], thereby effectively suppressing tumor proliferation, invasion, and metastasis. However, most targeted agents only inhibit malignant biological behaviors rather than directly killing tumor cells. Among various therapeutic targets, uPA has been well established as a critical mediator of CRC invasion, metastasis, and poor prognosis [17,18,19,20,21]. A number of uPA inhibitors have been developed and evaluated in clinical trials; however, these agents show limited efficacy as monotherapy and require combination with chemotherapy to improve clinical benefits [22].

However, clinically available small-molecule uPA inhibitors display obvious limitations. Most exhibit poor target specificity and tend to inhibit other serine proteases including plasmin, thrombin, and factor Xa, which may cause off-target effects and bleeding complications [23]. As a typical cyclic peptide uPA inhibitor applied in this work, IG2 exerts anti-invasive and anti-metastatic effects by specifically recognizing and blocking the active site of uPA. Compared with small-molecule inhibitors, IG2 exhibits higher target selectivity and lower bleeding risk, which aligns with the advantages of peptide-based agents [24]. Peptide-based uPA inhibitors feature high specificity and favorable biosafety [24,25], yet their extremely short half-life and rapid renal clearance severely compromise in vivo efficacy and clinical translation [26]. Furthermore, traditional uPA-targeted therapy and chemotherapy have overlapping antitumor mechanisms, making it difficult to achieve ideal synergistic amplification. Therefore, the development of a novel combination strategy with complementary mechanisms is essential to overcome the bottlenecks of uPA-targeted therapy and PDT.

**Scheme 1:**
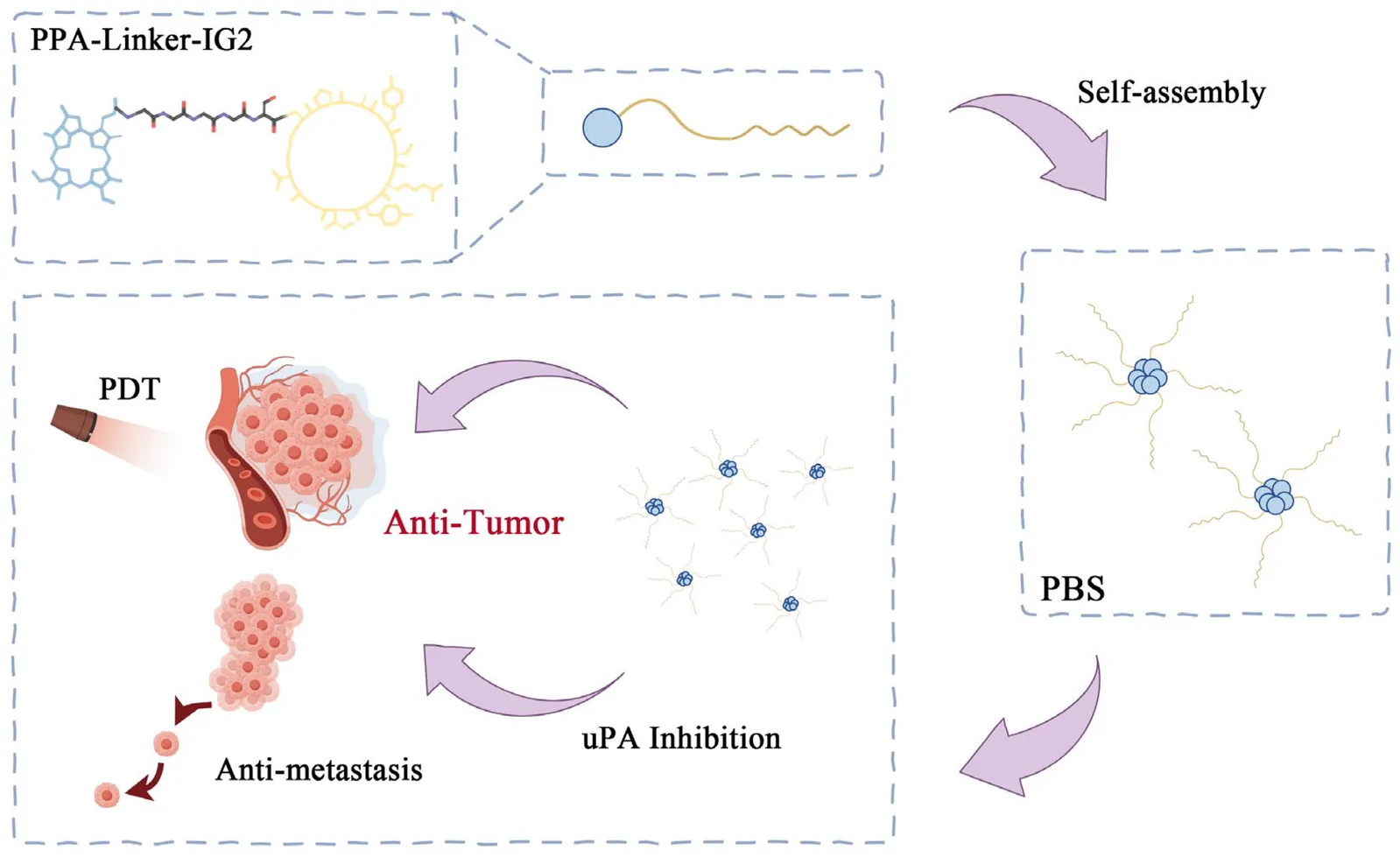
Schematic illustration of the construction and antitumor mechanism of PINPs. The amphiphilic conjugate PPA-Linker-IG2 self-assembles into uniform nanoparticles (PINPs) in PBS solution. After intravenous administration, PINPs accumulate in tumor tissues and exert dual functions: the photosensitizer PPA enables photodynamic therapy (PDT) to directly ablate primary tumors under light irradiation, while the IG2 peptide inhibits uPA activity to suppress tumor invasion and metastasis.

To address the above challenges, this study rationally integrates uPA-targeted therapy with PDT to construct a synergistic antitumor nanoplatform with complementary functions. On the basis of our previous research, we designed and chemically conjugated the photosensitizer PPA with the high-affinity uPA cyclic peptide inhibitor IG2 to generate an amphiphilic small-molecule conjugate (PPA-IG2), which spontaneously self-assembles into uniform nanoparticles denoted as PINPs (PPA-IG2 nanoparticles). This molecular design aims to combine the rapid tumoricidal capacity of PDT with the long-term anti-metastatic effect of uPA inhibition to achieve multidimensional synergistic therapy for CRC (Scheme 1).

This integrated design presents multiple unique advantages. The amphipathic PPA-IG2 conjugate spontaneously self-assembles into uniform spherical nanoparticles in aqueous solution driven by hydrophobic interactions and π–π stacking, which improves colloidal stability, prolongs blood circulation, and facilitates passive tumor targeting via the EPR effect [27,28,29]. Meanwhile, the IG2 peptide displayed on the nanoparticle surface can specifically recognize and bind uPA in the tumor microenvironment, directly exerting targeted protease inhibitory activity. Following cellular internalization, the PPA moiety generates abundant ROS under 680 nm laser irradiation to exert potent PDT effects for direct tumor ablation [30]. Simultaneously, the IG2 component sustains uPA inhibition to exert long-term suppression of tumor proliferation, invasion, and metastasis. Through the rational combination of targeted delivery, rapid photodynamic ablation, and durable anti-metastatic intervention, this nanosystem achieves remarkable synergistic therapeutic efficacy, offering a novel and clinically translatable strategy for efficient and precise treatment of colorectal cancer.

## 2. Materials and Methods

### 2.1. Materials and Reagents

All chemicals were purchased from Sigma-Aldrich (Shanghai, China) and used as received without further purification unless otherwise stated. The photosensitizer pyropheophorbide-a (PPA) was purchased from Yuanye Bio-Technology Co., Ltd. (Shanghai, China). The chromogenic substrate S-2444 for uPA activity assay was purchased from Chromogenix. Cell Counting Kit-8 (CCK-8) and reactive oxygen species (ROS) detection reagent DCFH-DA were obtained from Beyotime Biotechnology. All cell lines were purchased from the Shanghai Institute of Cell Biology, Chinese Academy of Sciences. Cells were cultured in Dulbecco’s modified Eagle medium (DMEM) (Hyclone, Logan, UT, USA) supplemented with 10% (*v*/*v*) fetal bovine serum (Gibco, Waltham, MA, USA). Male BALB/c mice (6–8 weeks old) were purchased from Shandong Pengyue Laboratory Animal Technology Co., Ltd. (Shandong, China). All animal experiments were approved by the Animal Ethics Committee of Shandong Second Medical University (No. 2025SDL268) and carried out in strict accordance with the relevant guidelines.

### 2.2. Synthesis and Characterization of PPA-IG2 Conjugate

PPA-IG2, Cy5-IG2, and the negative control conjugate PPA-R6A were all synthesized following the same conjugation strategy. Briefly, the IG2 peptide and its R6A mutant (with the 6th arginine residue substituted by alanine) were prepared by standard solid-phase peptide synthesis on Rink amide resin [31]. PPA with an activated carboxyl group and Cy5-NHS ester were each covalently coupled to the N-terminal amino group of the corresponding peptide via amide bond formation. All final products were purified by preparative high-performance liquid chromatography (HPLC) and characterized by ^1^H nuclear magnetic resonance (^1^H NMR) spectroscopy. This single-point R6A mutation has been previously validated to completely abolish the uPA inhibitory activity of the cyclic peptide, as this arginine is the key residue inserted into the S1 active pocket of uPA [24].

For nanoparticle preparation, PPA-IG2 was first dissolved in dimethyl sulfoxide (DMSO) and slowly added to phosphate-buffered saline (PBS, pH 7.4) under vigorous stirring to a final concentration of 1 mg/mL. The solution was stirred at room temperature for 2 h, followed by centrifugation at 10,000 rpm for 15 min to remove unassembled particles. The supernatant was lyophilized and stored at −20 °C until use.

The hydrodynamic diameter and polydispersity index (PDI) of PINPs were characterized using a dynamic light scattering instrument (DLS, Malvern Zetasizer Nano ZS, Malvern Panalytical, Malvern, UK) at 25 °C with a 2-min equilibration step. The morphology and size of the nanoparticles were further examined with a FlexSEM 1000II scanning electron microscope (Hitachi High-Tech, Tokyo, Japan): 10 μL of PINP solution was deposited onto a clean silicon wafer, air-dried at room temperature, and sputter-coated with a thin gold layer prior to imaging. Particle size quantification was performed using ImageJ 1.53t software (National Institutes of Health, Bethesda, MD, USA), with 100 individual particles randomly selected from multiple fields for statistical analysis.

For optical property and assembly state analysis, UV-Vis absorption and fluorescence spectra of PINPs in different media (PBS, DMSO, and 1 × 10^6^ cells/mL cell suspension) were acquired using a SpectraMax i3x multi-mode microplate reader (Molecular Devices, Sunnyvale, CA, USA); specifically, 5 μL of 400 μg/mL PINP stock solution was diluted with 95 μL of each corresponding medium to a final concentration of 10 μg/mL for all spectral measurements. For concentration-dependent absorption measurement, PINP stock solution was serially diluted with PBS to final concentrations of 10, 3.3, 1, 0.33, and 0.01 μg/mL, and spectra were collected under the same detection parameters.

### 2.3. ROS Quantum Yield Assay

The photodynamic effect of PINPs was evaluated by recording changes in the fluorescence signal of the fluorescent probe DCFH-DA. ROS generated by the photodynamic effect can oxidize DCFH-DA to form 2,7-dichlorofluorescein (DCF), which exhibits strong fluorescence. PINPs were diluted with cell suspension (1 × 10^6^ cells/mL) and PBS to a final concentration of 10 μg/mL, mixed with DCFH-DA at a final concentration of 10 μM. Irradiation was performed using a 680 nm LED light source (25 mW/cm^2^) placed directly above the 96-well plate. The fluorescence intensity (Ex/Em: 488/525 nm) was detected at intervals using a SpectraMax i3x multi-mode microplate reader: the sample was irradiated for 1 min and then immediately subjected to fluorescence measurement, and this irradiation–detection cycle was repeated continuously to track the time-dependent change in ROS level.

### 2.4. Enzyme Inhibition Activity Assay

The inhibitory activity of PINPs against uPA and other serine proteases (e.g., thrombin, trypsin) was determined using the chromogenic substrate method. Briefly, PINPs at various concentrations (0–10 μM) were incubated with uPA in Tris-HCl buffer (pH 7.4) at 37 °C for 10 min. After being irradiated or not irradiated with a 680 nm LED plate at 25 mW/cm^2^ for 5 min, the substrate S-2444 (Chromogenix, MA, USA) was added. The hydrolysis rate was recorded by monitoring the absorbance change at 405 nm over time. The inhibition constant (Ki) was calculated by fitting the data to the Morrison equation for tight-binding inhibition using GraphPad Prism 9.0 software (GraphPad Software, San Diego, CA, USA).

### 2.5. Cellular Uptake

CT26 murine colorectal cancer cells were used as the tumor model for efficacy evaluation owing to their high invasiveness and suitability for both in vitro invasion assays and in vivo metastasis studies, while human normal gastric mucosal epithelial GES-1 cells were employed as the normal control for biosafety assessment [1,32,33]. Mouse colorectal cancer cell line CT26 and human gastric mucosal cell line GES-1 were seeded in 6-well plates at a density of 2.5 × 10^5^ cells per well and cultured overnight to adhere. Cells were incubated with PINPs (10 μg/mL) for different time periods (1, 2, 4, 8, 24 h). Cells were then collected, and intracellular fluorescence intensity was analyzed using the APC channel (Ex 610 nm, Em 680 nm) of a flow cytometer (BD FACS Celesta, BD Biosciences, San Jose, CA, USA).

### 2.6. Subcellular Localization

Confocal laser scanning microscopy (Leica TCS SP8, Leica Microsystems, Wetzlar, Germany) was used to observe the intracellular localization of PINPs. Cells were seeded in confocal dishes at 2 × 10^5^ cells per dish and cultured overnight. Each dish was incubated with medium containing PINPs at 2 μg/mL for 4 h and then washed 3 times with PBS. Cells were incubated with 200 nM Mito-Tracker Green and 50 nM Lyso-Tracker Red at 37 °C for 45 min, respectively. After incubation, cells were washed 3 times with medium, fixed with 1 mL immunostationary fixative at room temperature for 10 min, and washed 3 times with PBS for further use. Before staining with DAPI, cells were fixed with 1 mL immunostationary fixative at room temperature for 10 min, washed with PBS, and then stained with 1 mL DAPI staining solution at room temperature for 20 min. Intracellular fluorescence of DAPI, Mito-Tracker Green, Lyso-Tracker Red, and PINPs was imaged using filters at Ex 340 nm/Em 460 nm, Ex 490 nm/Em 516 nm, Ex 577 nm/Em 590 nm, and Ex 610 nm/Em 677 nm, respectively.

### 2.7. In Vitro Cytotoxicity

The phototoxicity and dark toxicity of PINPs were evaluated using mouse colorectal cancer cell line CT26 and human gastric mucosal cell line GES-1. A volume of 100 μL cell suspension was added to 96-well plates to maintain 1 × 10^3^ cells per well. For dark toxicity evaluation, cells were incubated with free PPA, PINPs, or PPA-R6A at the high concentration of 200 μg/mL at 37 °C for 24 h. Then, 10 μL of CCK-8 solution was added, and cells were incubated for another 1 h. The absorbance at 450 nm was detected using a multifunctional microplate reader. For phototoxicity evaluation, cells were incubated with PINPs (0–10 μg/mL) for 4 h and then irradiated with a 680 nm LED light source at 25 mW/cm^2^ for 80 s with a light dose of 2 J/cm^2^. After further incubation for 24 h, cell viability was detected using CCK-8 assay. All experiments were repeated three times.

### 2.8. Cell Death Analysis

Cell death of CT26 cells after PINP treatment was analyzed using a flow cytometer (BD FACSAria III, BD Biosciences, San Jose, CA, USA). First, CT26 cells were seeded in 6-well plates at 2.5 × 10^5^ cells per well and cultured overnight at 37 °C to adhere. CT26 cells were incubated with or without PINPs (0.66 μg/mL) for 4 h. After incubation, cells were irradiated with a 680 nm LED light source at 25 mW/cm^2^ for 80 s with a light dose of 2 J/cm^2^. After further incubation at 37 °C for 4 h, cells were digested with EDTA-free trypsin and collected.

CT26 cells were resuspended in pre-cooled PBS according to the instructions of the Annexin V-PE/7-AAD kit. A volume of 100 μL cell suspension at 2 × 10^6^ cells/mL was centrifuged at 300 *g*, 4 °C for 5 min. The supernatant was discarded, and 5 μL Annexin V-PE, 10 μL 7-AAD staining solution, and 100 μL 1× Binding Buffer were added sequentially to resuspend the cells. After incubation at room temperature in the dark for 15 min, 200 μL of 1× Binding Buffer was added and mixed. Samples were placed on ice and detected by flow cytometry within 1 h. PE and 7-AAD were used as horizontal and vertical coordinates, respectively, to plot the double scatter plot.

### 2.9. Transwell Invasion Experiment

In the experiment of PINPs inhibiting tumor cell invasion, the upper chamber of a Transwell chamber (Corning Inc., Corning, NY, USA) was prepared strictly according to the instructions of BD Matrigel (Corning Inc., Corning, NY, USA) [34]. First, Matrigel was diluted 30-fold according to the batch concentration to a final concentration of 0.2–0.3 mg/mL. A volume of 100 μL diluted Matrigel was added to the upper chamber using a pre-cooled pipette tip and incubated at 37 °C for 1 h to solidify. CT26 cells were collected in FBS-free DMEM, and 100 μL of cell suspension containing approximately 3 × 10^4^ cells was added to the upper chamber. PINPs were diluted with FBS-free DMEM to final concentrations of 0–200 μg/mL, and 100 μL of the dilution was added to each well. Free PPA and PPA-R6A were set as negative controls and tested at a single final concentration of 200 μg/mL. PPA-R6A lacks uPA inhibitory activity due to the R6A point mutation while retaining the same physicochemical properties as PINPs, which helps exclude non-specific peptide-related effects. Then, 600 μL of complete DMEM containing 10% FBS was added to the lower chamber and cultured at 37 °C. After 24 h, the upper chamber solution was discarded, and cells inside the upper chamber membrane were carefully removed using a sterile cotton swab. The upper chamber was immersed in methanol solution for 20 min to fix cells that had migrated to the outside of the membrane. After air-drying completely, the upper chamber was immersed in 0.1% crystal violet solution for 20 min to stain migrated cells. After washing with PBS until colorless, cells were observed under a microscope. Finally, crystal violet was eluted with 33% acetic acid solution, and the absorbance at 570 nm was detected using a multifunctional microplate reader to calculate the invasion rate.

### 2.10. In Vivo Antitumor Effects

The antitumor effect of PINPs was evaluated by establishing a CT26 tumor-bearing mouse model. Approximately 2 × 10^6^ viable cells suspended in 100 μL sterile PBS were injected subcutaneously into the right flank of each male BALB/c mouse. Tumor growth was observed daily. When the tumor volume reached approximately 100 mm^3^ (about 3–4 days), mice were randomly divided into 5 groups (*n* = 6 per group). Saline, IG2 (1 mg/kg), and three groups of PINPs were injected via the tail vein. The saline group and IG2 group were administered once every 2 days. Among the three PINP treatment groups, one group was injected with 2 mg/kg PINPs (equivalent to 1 mg/kg IG2) every 2 days. The other two groups received only one injection of PINPs at concentrations of 2 mg/kg and 0.2 mg/kg, respectively. At 4 h post-injection, these two single-dose groups received 680 nm laser irradiation delivered by a dual-wavelength fiber laser (Xi’an Leize Electronic Technology Co., Ltd., Xi’an, China). The laser output was set at a power density of 400 mW/cm^2^ with an irradiation distance of 1 cm above the tumor surface, and the irradiation lasted for 120 s, corresponding to a total light dose of 48 J/cm^2^.The entire treatment period lasted 8 days, and mouse body weight and tumor volume were monitored daily. Tumor volume was calculated using the formula:

(1) $$V=\frac{AB^{2}}{2}$$

where *A* and *B* represent the maximum length and minimum width of the tumor measured with a vernier caliper, respectively.

Tumor tissues were photographed and sent for histopathological analysis after resection on Day 8.

### 2.11. In Vivo Anti-Metastatic Efficacy

A CT26 lung metastasis model was established based on male BALB/c mice to evaluate the anti-metastatic efficacy of PINPs in vivo. BALB/c mice weighing approximately 20 g were divided into 5 groups with 5 mice per group. The CT26 cell line containing the GFP gene was purchased from PerkinElmer (Waltham, MA, USA). A volume of 100 μL CT26-GFP cell suspension containing 1 × 10^7^ cells in saline was injected intravenously. One group without tumor implantation was set as a control. The four implanted groups were intravenously administered saline, 1 mg/kg IG2, 0.2 mg/kg PINPs, and 2 mg/kg PINPs, respectively. Drugs were injected via the tail vein once every 48 h. After continuous treatment for 12 days, mice were sacrificed by cervical dislocation, and lung tissues were collected. After weighing the lung tissues, half of the lung tissues were used for qPCR detection of exogenous GFP gene, and the other half were used for HE staining and Masson staining. The qPCR analysis was based on the amplification of a segment of GFP-DNA sequence that does not exist in mouse tissues. The amplified fragment was 287 bp, with primers 5′-CAGTGCTTCAGCCGCTAC-3′ (forward) and 5′-TTCACCTTGATGCCGTTC-3′ (reverse).

### 2.12. In Vivo Imaging and Tissue Distribution

CT26 tumor-bearing BALB/c mice were randomly divided into 2 groups (*n* = 5 per group) with an average body weight of approximately 20 g and average tumor volume of approximately 0.5 cm^3^. PINPs or Cy5-labeled IG2 (Cy5-IG2, as a non-self-assembled peptide control) was injected into mice via the tail vein at a dose of 2 mg/kg. Blood samples were collected from the retro-orbital plexus at different time points (2 min, 30 min, 4 h, 8 h, 24 h, 48 h). Blood was centrifuged to collect plasma. A volume of 50 μL plasma was mixed with 50 μL 10% SDS, and fluorescence was detected using a multifunctional microplate reader at channels Ex 610 nm/Em 680 nm and Ex 630 nm/Em 670 nm, respectively.

CT26 tumor-bearing BALB/c mice were randomly divided into 10 groups (*n* = 5 per group) with an average body weight of approximately 20 g and average tumor volume of approximately 0.5 cm^3^. PINPs or Cy5-labeled IG2 (Cy5-IG2, as a non-self-assembled peptide control) was injected into mice via the tail vein at a dose of 2 mg/kg. Mice were sacrificed at designated time points after injection (0.5 h, 3 h, 6 h, 24 h, 48 h), and major organs (heart, liver, spleen, lung, kidney) and tumors were collected. An in vivo imaging system (IVIS Spectrum, PerkinElmer) was used to analyze fluorescence signals of PPA or Cy5 in tissues and tumors at channels Ex 610 nm/Em 680 nm and Ex 630 nm/Em 670 nm, respectively.

### 2.13. Statistical Analysis

All data are expressed as mean ± standard deviation (SD). Statistical analysis of differences between groups was performed using GraphPad Prism 9.0 software (GraphPad Software, San Diego, CA, USA) by one-way or two-way analysis of variance (ANOVA). *p* < 0.05 was considered statistically significant.

## 3. Results

### 3.1. Synthesis and Physicochemical Characterization of PINPs

The PPA-IG2 conjugate was successfully synthesized by covalently linking PPA to the N-terminus of IG2 peptide through a flexible linker. The crude product was purified by preparative HPLC, and its chemical structure was confirmed by ^1^H NMR spectroscopy (Figures S1–S3). Under physiological conditions, the amphiphilic PPA-IG2 conjugate spontaneously self-assembled into uniform spherical nanoparticles (PINPs) driven by hydrophobic interactions and π-π stacking of the porphyrin rings.

**Figure 1:**
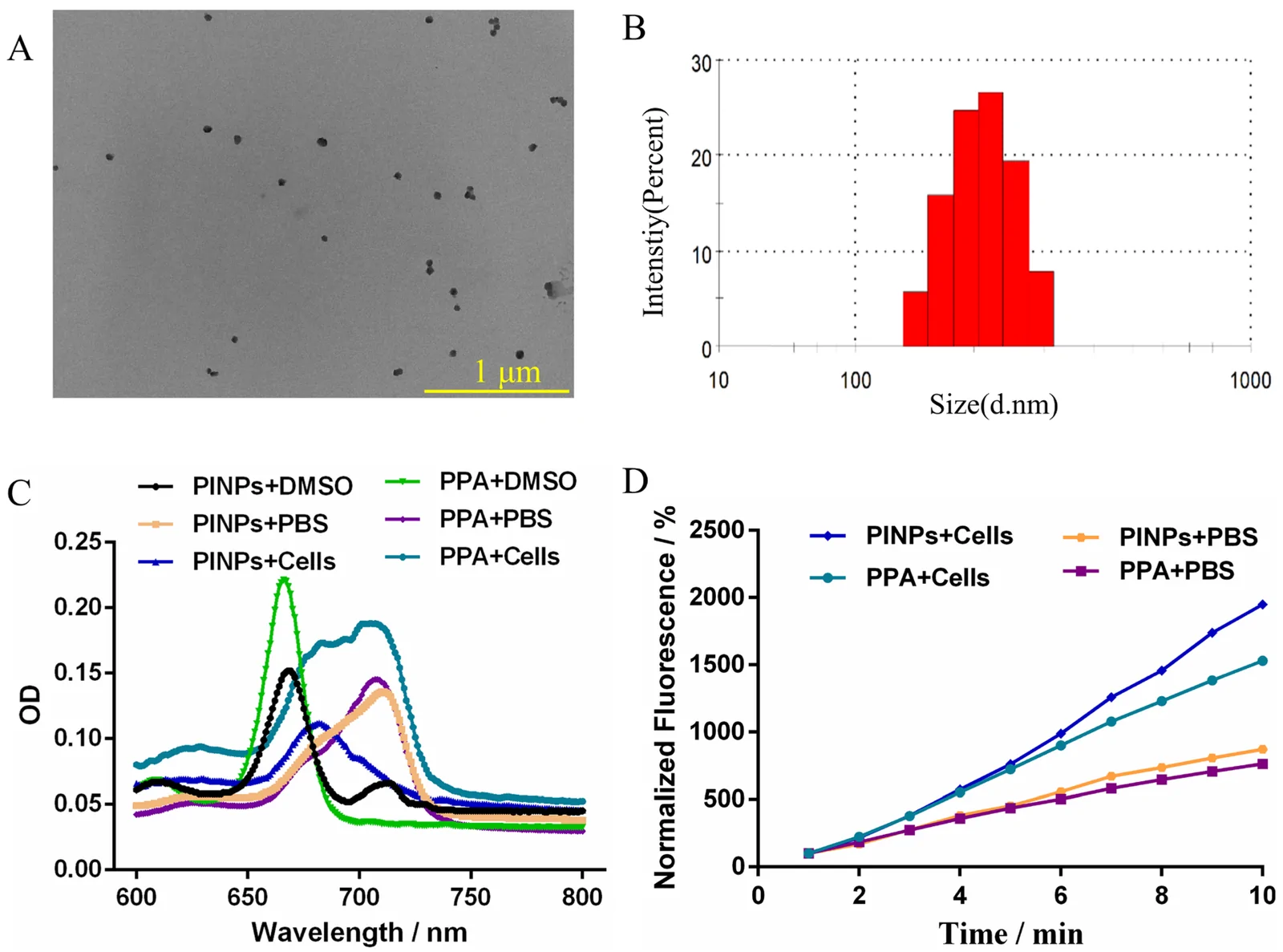
Characterization of PINPs. (**A**) Scanning electron microscopy image of PINPs. (**B**) Hydrodynamic diameter distribution of PINPs measured by dynamic light scattering. (**C**) UV-Vis absorption spectra of PPA and PINPs in different conditions. (**D**) Reactive oxygen species generation by PINPs under laser irradiation.

Scanning electron microscopy revealed that PINPs exhibited a well-defined spherical morphology with good dispersity. Quantitative analysis of 100 randomly selected particles from multiple fields using ImageJ software (National Institutes of Health, Bethesda, MD, USA) yielded an average dry diameter of 63.81 ± 11.15 nm (Figure 1A). Dynamic light scattering analysis showed that PINPs had an average hydrodynamic diameter of 208 ± 3 nm in PBS with a narrow polydispersity index (PDI) of 0.16 ± 0.01, indicating excellent colloidal stability and monodispersity (Figure 1B). This particle size is optimal for tumor accumulation via the enhanced permeability and retention (EPR) effect, as it facilitates extravasation through tumor vasculature while avoiding rapid renal clearance.

UV-Vis absorption spectroscopy was performed to characterize the optical properties of PPA-IG2 monomers and assembled PINPs. PPA-IG2 monomers dissolved in DMSO showed a maximum Q-band absorption peak at 667 nm, whereas spontaneously assembled PINPs in PBS exhibited a significantly red-shifted main peak at 710 nm (Figure 1C). Concentration-gradient measurements further showed that the absorption peak position remained stable at 710 nm across the tested range, with absorbance increasing proportionally with rising PINP concentration (Figure S4). After co-incubation with CT26 tumor cells, the main absorption peak of PINPs blue-shifted to approximately 680 nm and presented a single symmetric peak, indicating partial disassembly into monomeric/oligomeric states triggered by the lipid-rich cellular microenvironment. In contrast, free PPA showed a dominant aggregated-state peak at 710 nm with a distinct shoulder peak around 680 nm under the same incubation conditions, due to its poor aqueous solubility and spontaneous formation of irregular aggregates in the cell culture medium.

### 3.2. Photodynamic Activity of PINPs

The photodynamic therapeutic potential of PINPs was evaluated by measuring reactive oxygen species (ROS) generation under laser irradiation. Using the DCFH-DA (Beyotime Biotechnology, Shanghai, China) fluorescent probe, we found that PINPs exhibited excellent photodynamic activity upon 680 nm LED irradiation (25 mW/cm^2^). The fluorescence intensity of DCF increased by 7- to 20-fold after 10 min of irradiation, indicating robust ROS production (Figure 1D).

Notably, PINPs dispersed in cell suspension generated significantly higher levels of ROS compared to those in PBS alone. This enhanced photodynamic activity in the cellular environment can be attributed to the favorable lipid environment of cell membranes, which promotes partial disassembly of PINPs into monomeric units, optimizes the photophysical properties of PPA, and facilitates ROS generation. This characteristic is particularly advantageous for tumor therapy, as it ensures efficient ROS generation specifically within the tumor microenvironment.

### 3.3. uPA Inhibitory Activity of PINPs

**Figure 2:**
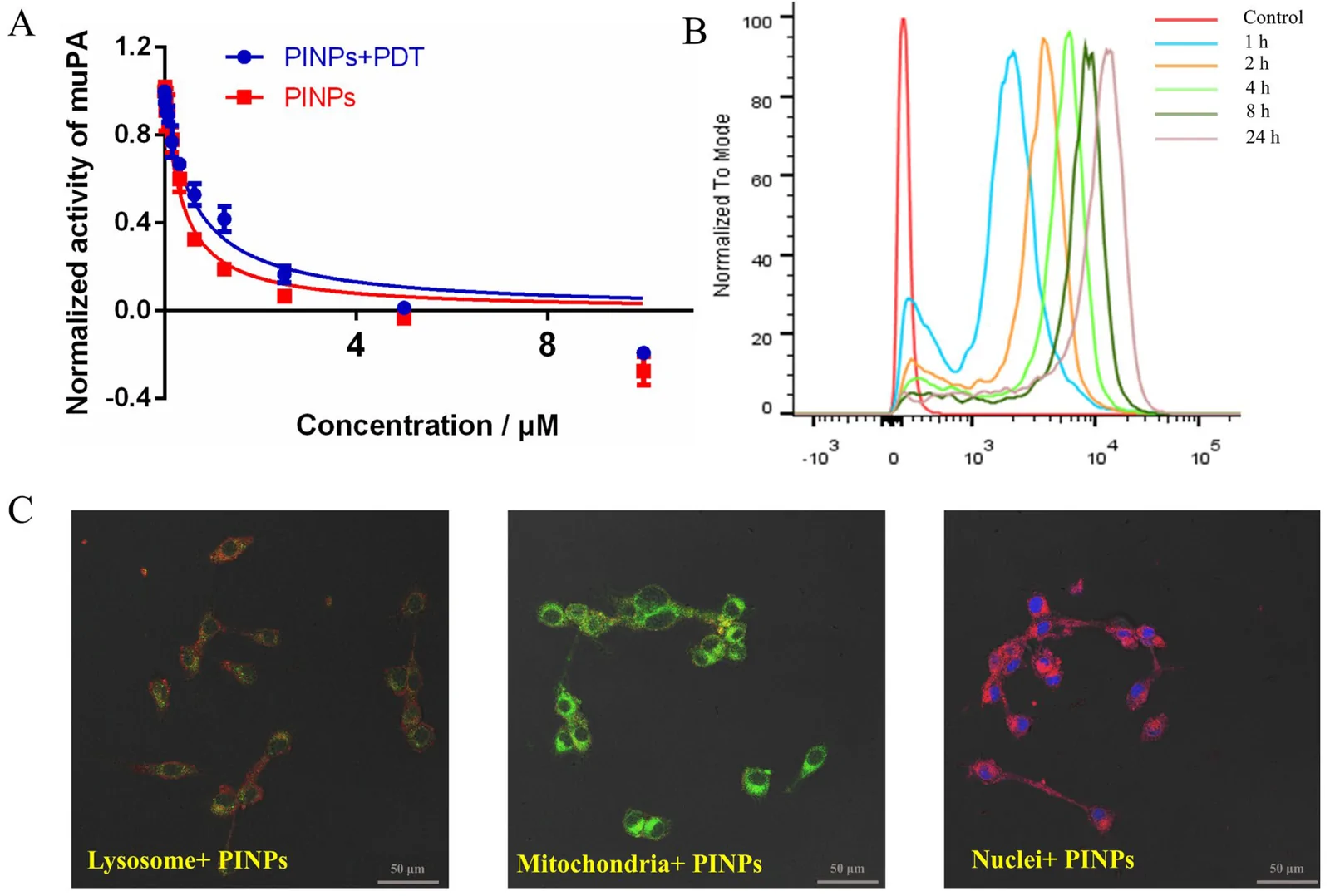
uPA inhibition and cellular uptake of PINPs. (**A**) Inhibition constants of PINPs against uPA determined by chromogenic substrate assay. (**B**) Time-dependent cellular uptake of PINPs in CT26 cells quantified by intracellular fluorescence intensity measurement. (**C**) Subcellular localization of PINPs in CT26 cells after 4 h incubation with 2 μg/mL PINPs. Lysosomes were labeled with Lyso-Tracker Red (Ex/Em: 577/590 nm), mitochondria were labeled with Mito-Tracker Green (Ex/Em: 490/516 nm), and nuclei were counterstained with DAPI (Ex/Em: 340/460 nm). The intrinsic fluorescence of PPA was detected at Ex/Em: 610/677 nm.

The ability of PINPs to inhibit uPA enzymatic activity was assessed using chromogenic substrate assays. PINPs exhibited potent and concentration-dependent inhibitory activity against uPA with a *Ki* value of 0.328 ± 0.055 μM, indicating high-affinity binding to the target enzyme (Figure 2A). This inhibitory potency is comparable to that of the free IG2 peptide, confirming that conjugation to PPA and self-assembly do not compromise the target recognition capability of IG2.

A critical concern for peptide-based photosensitizer conjugates is whether ROS generated during PDT might oxidize and inactivate the peptide component. To address this, we evaluated the uPA inhibitory activity of PINPs after light irradiation. Remarkably, PINPs maintained significant inhibitory activity (*Ki* = 0.603 ± 0.082 μM) even after exposure to 680 nm irradiation (25 mW/cm^2^, 5 min). This stability demonstrates that the flexible linker effectively separates the photodynamic module from the targeting peptide, preserving the biological activity of IG2 during PDT. This dual functionality ensures simultaneous anti-metastatic and photodynamic therapeutic effects.

### 3.4. Cellular Uptake and Subcellular Localization

Cellular internalization of PINPs was investigated in CT26 colorectal cancer cells and GES-1 normal gastric mucosal cells using flow cytometry (Figure 2B and Figure S5). PINPs were efficiently internalized by CT26 cells in a time-dependent manner, with uptake increasing steadily over 24 h. Compared to normal GES-1 cells, CT26 cancer cells exhibited significantly higher uptake efficiency, indicating preferential accumulation in tumor cells. Free PPA showed faster cellular uptake, reaching saturation within 2 h. In contrast, PINPs exhibited gradual and sustained internalization. However, cellular uptake of PINPs requires a longer time to reach saturation, presumably because the IG2 peptide exposed on the nanoparticle surface can specifically bind to uPA in the extracellular matrix, thereby retaining PINPs outside the cell temporarily and delaying the rate of internalization.

Confocal laser scanning microscopy revealed that PINPs primarily localized in mitochondria and lysosomes of CT26 cells, with minimal nuclear entry (Figure 2C). This subcellular distribution is strategically advantageous for PDT, as mitochondria are key organelles involved in apoptosis induction, and lysosomal disruption can effectively trigger cell death pathways while minimizing the risk of genomic damage.

### 3.5. In Vitro Anti-Tumor and Anti-Invasive Effects

**Figure 3:**
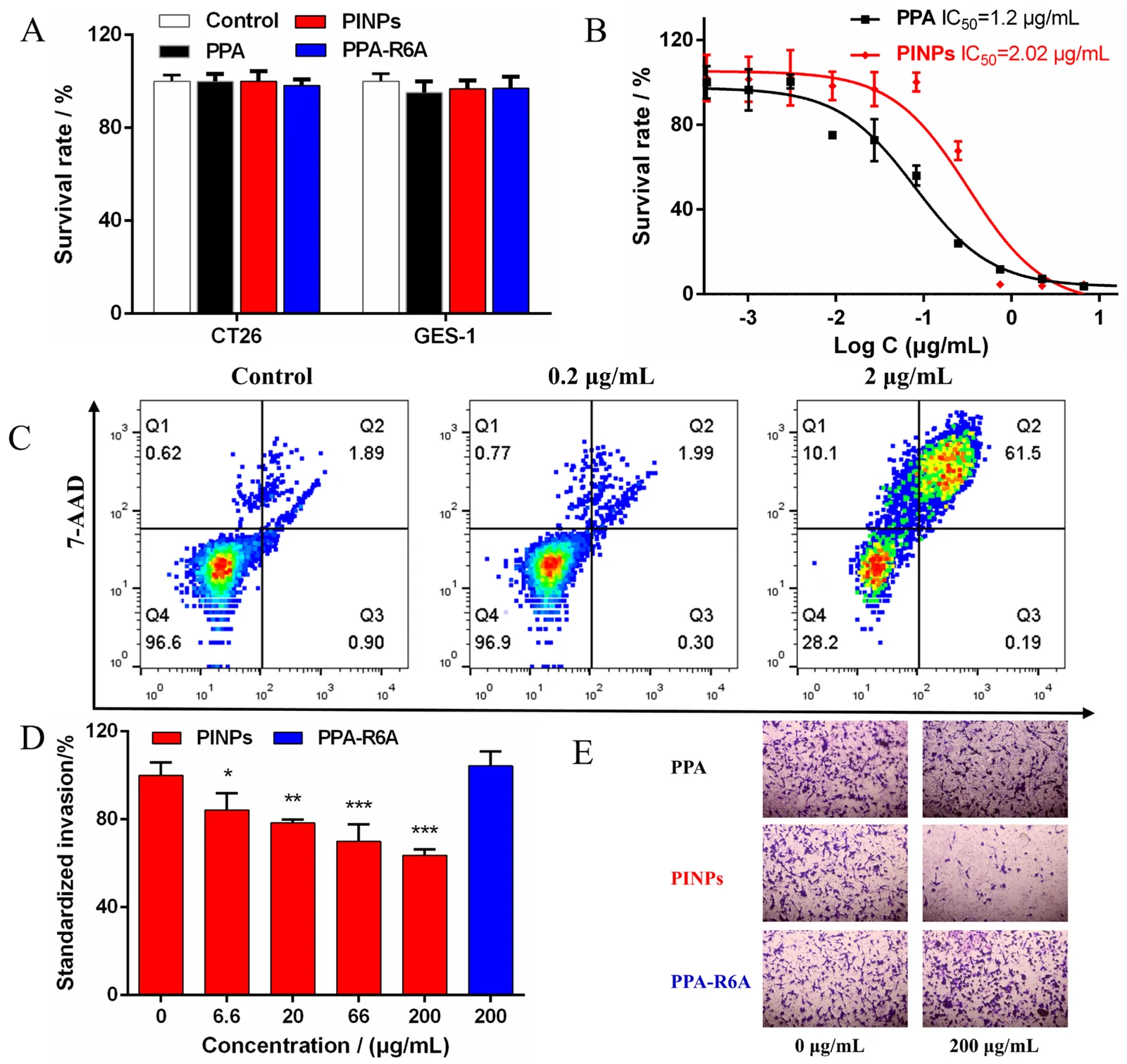
In vitro antitumor and anti-invasive effects of PINPs. (**A**) Dark toxicity of free PPA, PINPs, and PPA-R6A against CT26 and GES-1 cells after 24 h incubation at 200 μg/mL. (**B**) Phototoxicity of PINPs against CT26 cells. Cells were incubated with PINPs (0–20 μg/mL) for 4 h, then irradiated with 680 nm LED light (25 mW/cm^2^, 80 s, total light dose 2 J/cm^2^). (**C**) Cell death induction by PINPs with PDT. Apoptosis was analyzed by flow cytometry using Annexin V-PE/7-AAD double staining. Cells were incubated with 0.66 μg/mL PINPs for 4 h, irradiated with 680 nm light (25 mW/cm^2^, 2 J/cm^2^), and detected after 4 h further incubation. (**D**) Quantitative analysis of CT26 cell invasion inhibition. PINPs were tested at a concentration gradient of 0–200 μg/mL, and the uPA-inactive control PPA-R6A was tested at a single concentration of 200 μg/mL. (**E**) Representative microscopic images of invaded CT26 cells in the control, free PPA, PINPs, and PPA-R6A groups (all treated at 200 μg/mL). Values are represented as mean ± SD from three independent biological experiments. * p < 0.05, ** p < 0.01, *** p < 0.001 vs. the vehicle group.

The in vitro therapeutic efficacy of PINPs was evaluated by assessing cytotoxicity and anti-invasive activity. In the absence of light irradiation (dark toxicity), free PPA, PINPs, and the uPA-inactive control PPA-R6A all showed no significant cytotoxicity against both CT26 and GES-1 cells at 200 μg/mL, demonstrating excellent biocompatibility and ruling out non-specific dark cytotoxic artifacts at the tested concentration (Figure 3A). Upon 680 nm laser irradiation, both PINPs and PPA exhibited potent phototoxicity against CT26 cells, with IC_50_ values of 2.02 μg/mL and 1.2 μg/mL, respectively (Figure 3B). Cell death analysis confirmed that PINPs plus laser irradiation effectively induced apoptosis in CT26 cells (Figure 3C). The combination of targeted delivery and precise light activation ensures minimal damage to normal cells while maximizing tumor cell killing.

The anti-invasive activity of PINPs was evaluated using Transwell invasion assays (Figure 3D,E). PINPs significantly inhibited the invasion of CT26 cells in a concentration-dependent manner, achieving 36.4% inhibition at 200 μg/mL. In contrast, neither free PPA nor the uPA-inactive control PPA-R6A showed detectable anti-invasive activity at 200 μg/mL, confirming that the inhibition of invasion specifically results from uPA blockade by the intact IG2 sequence, rather than non-specific effects of the photosensitizer backbone or peptide scaffold. These results demonstrate that PINPs exert dual anti-tumor effects: specific inhibition of tumor cell invasion through uPA targeting and light-controlled tumor cell ablation via PDT.

### 3.6. Prolonged Circulation and Enhanced Tumor Accumulation

A major limitation of free IG2 peptide is its extremely short half-life due to rapid renal clearance. To evaluate whether self-assembly into nanoparticles could overcome this limitation, we investigated the in vivo pharmacokinetics and biodistribution of PINPs compared to Cy5-labeled IG2 (Cy5-IG2).

**Figure 4:**
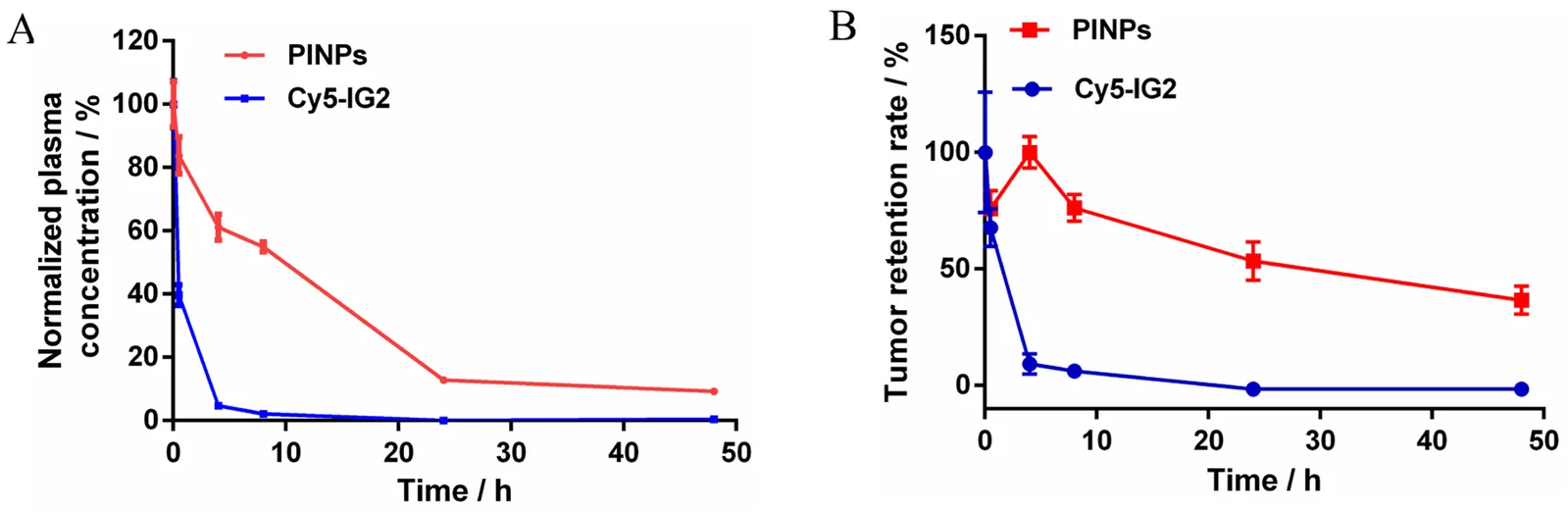
In vivo pharmacokinetics and tumor accumulation of PINPs. (**A**) Blood circulation profiles of Cy5-IG2 and PINPs in CT26 tumor-bearing BALB/c mice after intravenous injection at a dose of 2 mg/kg. Plasma fluorescence intensity was quantified at preset time points. (**B**) Tumor retention of Cy5-IG2 and PINPs over time in CT26 tumor-bearing mice, detected by ex vivo fluorescence imaging at designated time points post-injection. Data are presented as mean ± SD (*n* = 5).

Pharmacokinetic analysis revealed that PINPs exhibited dramatically prolonged blood circulation compared to the free peptide (Figure 4A). Cy5-IG2 was rapidly cleared from the bloodstream, with 60.4% eliminated within 30 min and almost complete clearance by 8 h (only 2.1% remaining). In striking contrast, PINPs maintained 54.9% of the initial dose in circulation after 8 h and remained detectable even at 48 h post-administration. This represents a greater than 20-fold extension in half-life, effectively addressing the critical pharmacokinetic limitation of small peptide therapeutics.

Ex vivo fluorescence imaging of tumors and major organs confirmed efficient tumor accumulation of PINPs (Figure S6). PINPs rapidly accumulated in tumor tissues, reaching maximum levels within 4 h and maintaining significant retention for up to 48 h (36.6% of peak signal). In comparison, Cy5-IG2 showed minimal tumor accumulation and was rapidly cleared from all tissues. The superior tumor retention of PINPs can be attributed to the EPR effect and nanoparticle stability, ensuring prolonged exposure of the tumor to therapeutic concentrations.

### 3.7. In Vivo Antitumor Efficacy

**Figure 5:**
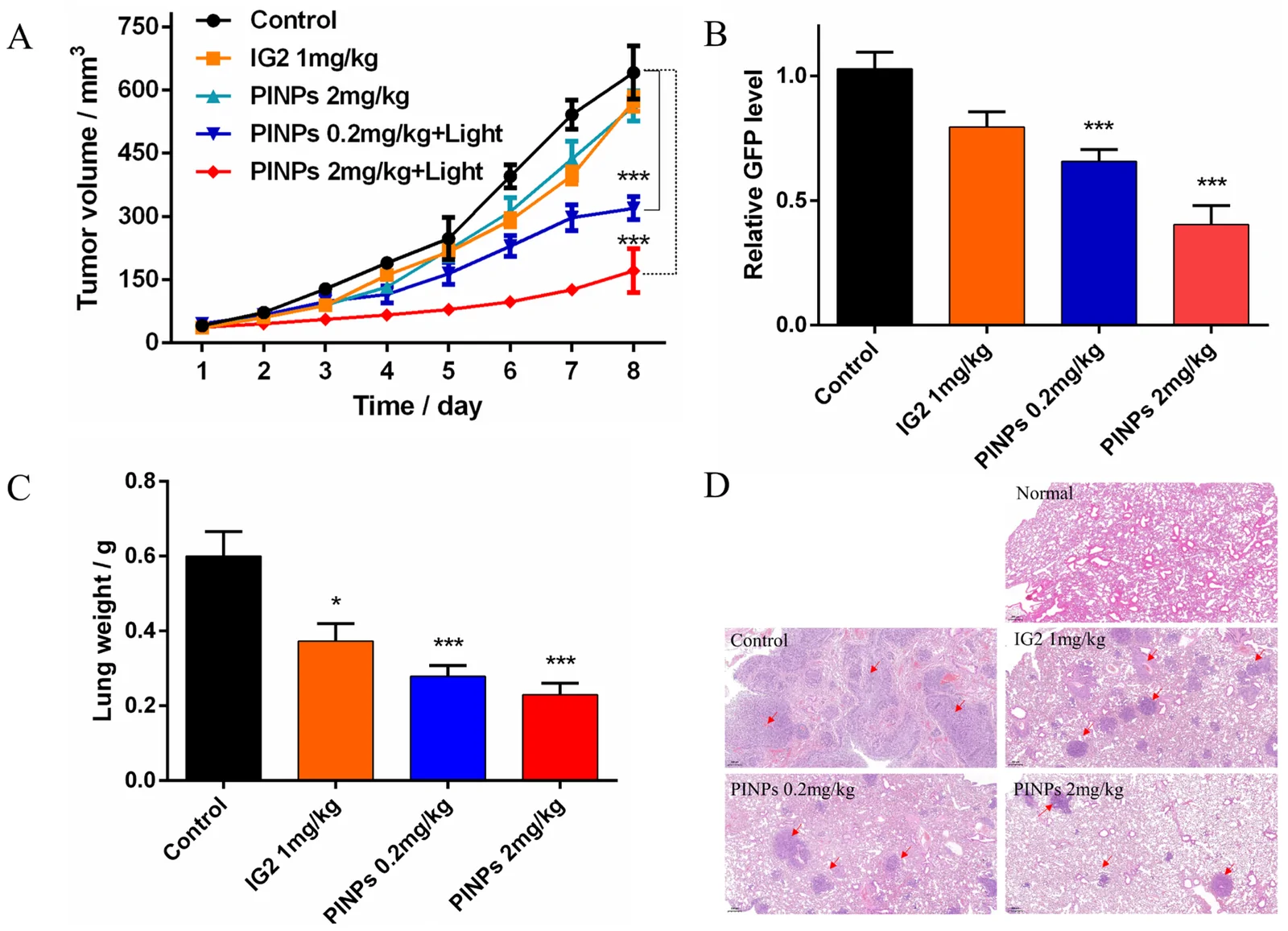
In vivo antitumor and anti-metastatic efficacy of PINPs. (**A**) Tumor growth inhibition in subcutaneous xenograft model. CT26 tumor-bearing mice received different treatment regimens, with 680 nm laser irradiation (400 mW/cm^2^, 120 s, total light dose 48 J/cm^2^) at 4 h post-injection. Data are presented as mean ± SD (*n* = 6). (**B**) Quantitative analysis of lung metastasis by GFP qPCR. CT26 cells were stably transfected with exogenous GFP gene, which is absent in normal mouse tissues; thus, the GFP DNA level in lung tissue directly reflects the burden of metastatic tumor cells. Mice received intravenous administration every 48 h for 12 days. Data are presented as mean ± SD (*n* = 5). (**C**) Lung weight measurement of metastatic model. (**D**) Histological analysis of lung tissues with HE staining. * *p* < 0.05, *** *p* < 0.001 vs. the vehicle group.

The in vivo therapeutic potential of PINPs was evaluated in CT26 subcutaneous xenograft models. Tumor-bearing mice were treated with saline, free IG2, or PINPs with or without PDT (Figure 5A and Figure S7A). Consistent with previous reports, multiple doses of IG2 can inhibit tumor growth; however, in this study, single-dose administration of IG2 showed limited therapeutic effect. Similarly, non-irradiated PINPs did not significantly inhibit tumor growth, confirming the requirement for light activation to achieve therapeutic efficacy.

In contrast, PINPs combined with PDT exhibited remarkable tumor growth inhibition. Both low-dose (0.2 mg/kg) and high-dose (2 mg/kg) PINPs plus laser irradiation achieved significant tumor suppression, with inhibition rates of 50.5% and 73.6%, respectively. The high-dose treatment group showed particularly potent antitumor efficacy, resulting in significant tumor regression. Importantly, all treatment groups maintained stable body weight throughout the experiment (Figure S7B), indicating excellent systemic safety without obvious adverse effects.

### 3.8. In Vivo Anti-Metastatic Activity

The anti-metastatic efficacy of PINPs was assessed in a CT26 lung metastasis model using GFP-labeled tumor cells for quantitative detection. Mice were treated with saline, IG2, or PINPs every 48 h for 12 days. Metastatic burden was evaluated by qPCR analysis of GFP DNA, lung weight measurement, and histological examination (Figure 5B–D).

qPCR analysis revealed that all treatment groups significantly reduced tumor metastasis compared to the saline control (Figure 5B). Free IG2 inhibited lung metastasis by 22.7%, consistent with its known anti-metastatic activity. PINPs exhibited significantly superior anti-metastatic efficacy, with 36.0% inhibition at low dose and an impressive 60.7% inhibition at high dose.

Lung weight measurements confirmed these results (Figure 5C). Metastatic lungs from the saline group were significantly heavier than normal lungs due to extensive tumor growth. Treatment with IG2 slightly reduced lung weight, but the difference was not statistically significant. In contrast, PINP treatment resulted in dramatic reductions in lung weight of 53.5% (low dose) and 61.7% (high dose), suggesting a potential inhibitory effect on metastatic tumor growth.

Histological analysis visually confirmed the anti-metastatic effects (Figure 5D). Saline-treated lungs showed extensive tumor nodules and tissue destruction. IG2 treatment moderately reduced metastatic burden, while PINP treatment almost completely eliminated metastatic lesions, with the high-dose group showing near-normal lung architecture. These results demonstrate that PINPs exert superior anti-metastatic activity compared to free IG2, attributed to improved pharmacokinetics and sustained uPA inhibition.

## 4. Discussion

Colorectal cancer remains a leading cause of cancer-related death worldwide, mainly due to its high invasiveness, frequent metastasis, and unsatisfactory efficacy of conventional treatments. Although photodynamic therapy (PDT) provides a minimally invasive and spatially controlled strategy for local tumor ablation, its clinical application is limited by insufficient tumor targeting, short action duration, and poor anti-metastatic potential [35]. Meanwhile, urokinase-type plasminogen activator (uPA) has been confirmed as a key driver of tumor invasion and metastasis [36], but peptide-based uPA inhibitors suffer from rapid blood clearance and poor tumor retention, resulting in unsatisfactory in vivo efficacy [24]. Therefore, the development of a nanoplatform that integrates PDT and sustained uPA inhibition to achieve synergistic tumor killing and anti-metastatic effects is highly desirable for advanced colorectal cancer therapy.

In this study, we successfully constructed a dual-functional self-assembled nanoparticle (PINPs) by conjugating pyropheophorbide-a (PPA) with IG2, a cyclic peptide with potent uPA inhibitory activity (Figure S1). Benefiting from the hydrophobic interaction and π–π stacking of PPA, the amphiphilic small-molecule conjugate could spontaneously self-assemble into uniform nanoparticles (Figure 1A,B) in aqueous solution [37]. Such a nanoplatform effectively prolonged blood circulation time and enhanced tumor accumulation via the EPR effect (Figure 4), which overcame the pharmacokinetic defects of small peptide inhibitors and improved local tumor retention. Notably, the particle size measured by SEM was smaller than the hydrodynamic diameter detected by DLS. This is a well-recognized phenomenon in nanoparticle characterization, as SEM measures the dry solid core of nanoparticles under high vacuum while DLS reflects the full hydrated size in aqueous solution, including the surface hydration layer and stretched surface peptide chains. The π–π stacking-driven assembly mechanism was further verified by concentration-dependent red-shifted absorption spectra (Figure S4) [38], consistent with the typical spectral features of porphyrin J-aggregates.

Benefiting from the rational molecular design with a GGGGS flexible linker, PINPs maintained two independent and complementary therapeutic functions. The flexible peptide linker effectively eliminates steric hindrance between the hydrophobic PPA moiety and the cyclic IG2 peptide, preserving the intact conformational freedom and target binding affinity of IG2 [39,40]. On one hand, PINPs could efficiently generate reactive oxygen species under 680 nm laser irradiation (Figure 1D) to induce tumor cell apoptosis, achieving precise and controllable photodynamic tumor ablation. This favorable photodynamic activity is closely related to the lipid-responsive disassembly property of PINPs: when nanoparticles come into contact with lipid components of cell membranes, the hydrophobic porphyrin moieties of PPA interact with lipid molecules, which disrupts the intermolecular π–π stacking and hydrophobic interactions within nanoparticles and triggers partial disassembly into monomers. The relief of PPA self-quenching in the assembled state further improves photophysical performance and significantly amplifies reactive oxygen species production [41,42]. Notably, free PPA tends to form non-specific aggregates in aqueous media with severe self-quenching, while the assembled-then-disassembled behavior of PINPs effectively avoids this drawback and achieves more efficient PDT activation in tumor cells. On the other hand, PINPs retained high uPA inhibitory activity (Figure 2A), which continuously suppressed tumor cell invasion and distant metastasis. More importantly, the photodynamic process did not impair the activity of the peptide segment, ensuring stable and long-term anti-metastatic efficacy during combination therapy.

In vitro experiments confirmed that PINPs exhibited negligible dark toxicity but remarkable phototoxicity against colorectal cancer cells, showing favorable tumor selectivity (Figure 3A,B). Transwell assays verified that PINPs significantly inhibited the invasive ability of CT26 cells in a concentration-dependent manner (Figure 3D,E), which was attributed to the specific uPA inhibition rather than non-specific cytotoxicity [24]. In vivo, PINPs exhibited excellent tumor accumulation and long-term retention compared with free peptide (Figure 4B). In subcutaneous tumor models, PINPs combined with PDT showed significant tumor growth inhibition (Figure 5A). In lung metastasis models, PINPs exerted superior anti-metastatic efficacy by sustained uPA suppression, which was significantly better than that of free IG2 peptide (Figure 5B–D). Throughout the treatment period, no obvious systemic toxicity or body weight loss was observed (Figure S7B), indicating favorable biosafety of PINPs.

Notably, this study has several limitations in terms of translational relevance. The IG2 cyclic peptide exhibits distinct species selectivity for uPA, and all in vivo experiments were conducted in the CT26 murine syngeneic model, which cannot fully recapitulate the genotypic heterogeneity of clinical colorectal cancer. Accordingly, the findings serve as proof-of-concept evidence rather than a direct prediction of clinical outcomes. Meanwhile, the 8-day subcutaneous tumor study was designed as a short-term acute tumor growth delay experiment adapted to rapid tumor proliferation and ethical requirements, and the observed effect should not be extrapolated as long-term curative efficacy. The 12-day lung metastasis model was used to evaluate anti-metastatic potential under sustained drug exposure, and long-term survival studies are warranted in future work. The core contribution of this work lies in proof-of-concept validation of the carrier-free dual-functional self-assembly strategy, and follow-up studies will optimize the peptide ligand for human uPA affinity and expand validation in more clinically relevant models [6].

In addition, there is room for further optimization of the experimental regimen. The single 4-h post-injection irradiation protocol was a procedural choice based on peak tumor accumulation, rather than a fully optimized regimen. Later irradiation time points and fractionated laser exposure may achieve a higher therapeutic index and alleviate PDT-induced transient oxygen depletion, which will be systematically explored in follow-up studies. The approximately 100-fold concentration difference between in vitro PDT cytotoxicity and anti-invasive activity stems from the distinct activation thresholds of ROS-mediated direct killing and protease inhibition-mediated phenotypic regulation. This gap does not negate the translational value of the dual-functional design, as the in vivo repeated dosing regimen can support sustained uPA inhibitory efficacy through cumulative tumor microenvironment modulation [22,24].

Collectively, this self-assembled nanoplatform successfully integrated photodynamic therapy and selective uPA inhibition, achieving synergistic tumor ablation and anti-metastasis effects against colorectal cancer. This strategy not only improves the pharmacokinetic performance of peptide agents but also realizes the complementary advantages of multiple therapeutic modalities, offering a promising design paradigm for the precise treatment of metastatic colorectal cancer.

## 5. Conclusions

In summary, we developed a dual-functional self-assembled nanoplatform (PINPs) by conjugating pyropheophorbide-a with a uPA-targeted cyclic peptide. PINPs spontaneously formed uniform nanoparticles under physiological conditions, which significantly prolonged blood circulation and enhanced tumor accumulation via the EPR effect. Upon laser irradiation, PINPs efficiently generated reactive oxygen species to achieve precise photodynamic tumor ablation. Meanwhile, PINPs maintained potent and sustained uPA inhibitory activity to suppress tumor invasion and metastasis. Within the tested observation windows, PINPs showed favorable synergistic therapeutic efficacy with excellent biosafety in both the subcutaneous tumor growth delay model and the lung metastasis model of colorectal cancer. This integrated strategy provides proof-of-concept validation for the synergistic anti-metastatic treatment of colorectal cancer, and also offers a generalizable design paradigm for the construction of peptide-based multifunctional nanotherapeutics.

## Data Availability

The original contributions presented in this study are included in the article/Supplementary Material. Further inquiries can be directed to the corresponding authors.

## Supplementary Materials

The following supporting information can be downloaded at: https://www.mdpi.com/article/10.3390/pharmaceutics18080948/s1. Figure S1. Chemical structure of the PPA-IG2 conjugate; Figure S2: HPLC chromatography of PPA-IG2; Figure S3: Mass spectrum of PPA-IG2; Figure S4: Concentration-dependent UV-Vis absorption spectra of PINPs; Figure S5: Time-dependent cellular uptake of PINPs in normal GES-1 cells and tumor CT26 cells; Figure S6: Ex vivo fluorescence imaging of tumors and major organs at different time points post-injection; Figure S7: Photographs of excised tumors and body weight changes of mice in different treatment groups.

## Abbreviations

The following abbreviations are used in this manuscript:

CRC	Colorectal cancer
PDT	Photodynamic therapy
uPA	Urokinase-type plasminogen activator
PPA	Pyropheophorbide-a
ROS	Reactive oxygen species
TAAs	Tumor-associated antigens
EPR	Enhanced permeability and retention effect

## References

[1] Frick C., Rumgay H., Vignat J., Ginsburg O., Nolte E., Bray F., Soerjomataram I. (2023). Quantitative estimates of preventable and treatable deaths from 36 cancers worldwide: A population-based study. Lancet Glob. Health.

[2] Morgan E., Arnold M., Gini A., Lorenzoni V., Cabasag C.J., Laversanne M., Vignat J., Ferlay J., Murphy N., Bray F. (2023). Global burden of colorectal cancer in 2020 and 2040: Incidence and mortality estimates from GLOBOCAN. Gut.

[3] Shinji S., Yamada T., Matsuda A., Sonoda H., Ohta R., Iwai T., Takeda K., Yonaga K., Masuda Y., Yoshida H. (2022). Recent Advances in the Treatment of Colorectal Cancer: A Review. J. Nippon. Med. Sch..

[4] Ni Y.F., Liang Y., Li M.Z., Lin Y., Zou X., Han F.Y., Cao J.N., Li L. (2023). The updates on metastatic mechanism and treatment of colorectal cancer. Pathol. Res. Pract..

[5] Shin A.E., Giancotti F.G., Rustgi A.K. (2023). Metastatic colorectal cancer: Mechanisms and emerging therapeutics. Trends Pharmacol. Sci..

[6] Pierre A., Favory R., Lancel S., Preau S. (2025). Can patient-derived in vitro models improve clinical translation in critical care research when used before animal studies?. Intensive Care Med. Exp..

[7] Sun H., Sklavenitis-Pistofidis R., Liu S.R., Liu X., Tsakmaklis N., Hatcher J.M., Guerrera M.L., Kofides A., Ramirez-Gamero A., Peachey A.L. (2026). Evolution of tumor subclones and T-cell dynamics underlie variable ibrutinib responses in Waldenström macroglobulinemia. Blood.

[8] Janas K., Boniewska-Bernacka E., Dyrda G., Slota R. (2021). Porphyrin and phthalocyanine photosensitizers designed for targeted photodynamic therapy of colorectal cancer. Bioorg. Med. Chem..

[9] Gu B.H., Wang B.F., Li X.M., Feng Z.D., Ma C.H., Gao L., Yu Y., Zhang J., Zheng P., Wang Y.P. (2022). Photodynamic therapy improves the clinical efficacy of advanced colorectal cancer and recruits immune cells into the tumor immune microenvironment. Front. Immunol..

[10] Chai Y., Xu M.X., Sun Y., Zhu Y.Y., Du T.Y., Zhu B.J., Yu H., Sun X.L., Pan Y., Liu Y. (2025). Hydrogen sulfide-responsive and depleting NIR-II nanoplatform synergistic photodynamic therapy for colorectal cancer. Chem. Eng. J..

[11] Sahoo B.M., Banik B.K., Borah P., Jain A. (2022). Reactive Oxygen Species (ROS): Key Components in Cancer Therapies. Anti-Cancer Agents Med. Chem..

[12] Chaturvedi P.K., Kim Y.W., Kim S.S., Ahn W.S. (2014). Phototoxic effects of pyropheophorbide-a from chlorophyll-a on cervical cancer cells. J. Porphyr. Phthalocyanines.

[13] Jiang W., Liang M., Lei Q., Li G., Wu S. (2023). The current status of photodynamic therapy in cancer treatment. Cancers.

[14] Alvarez N., Sevilla A. (2024). Current advances in photodynamic therapy (PDT) and the future potential of PDT-combinatorial cancer therapies. Int. J. Mol. Sci..

[15] Lu L., Jiang J., Zhan M., Zhang H., Wang Q.-T., Sun S.-N., Guo X.-K., Yin H., Wei Y., Li S.-Y. (2021). Targeting Tumor-Associated Antigens in Hepatocellular Carcinoma for Immunotherapy: Past Pitfalls and Future Strategies. Hepatology.

[16] Zhu G., Zhang Q., Zhang J., Liu F. (2021). Targeting tumor-associated antigen: A promising CAR-T therapeutic strategy for glioblastoma treatment. Front. Pharmacol..

[17] Berger D.H. (2002). Plasmin/Plasminogen System in Colorectal Cancer. World J. Surg..

[18] Zhuo J.L., Tan E.H., Yan B., Tochhawng L., Jayapal M., Koh S., Tay H.K., Maciver S.K., Hooi S.C., Salto-Tellez M. (2012). Gelsolin Induces Colorectal Tumor Cell Invasion via Modulation of the Urokinase-Type Plasminogen Activator Cascade. PLoS ONE.

[19] Ismail A.A., Shaker B.T., Bajou K. (2021). The Plasminogen–Activator Plasmin System in Physiological and Pathophysiological Angiogenesis. Int. J. Mol. Sci..

[20] Piet M., Paduch R. (2022). Ursolic and oleanolic acids in combination therapy inhibit migration of colon cancer cells through down-regulation of the uPA/uPAR-dependent MMPs pathway. Chem.-Biol. Interact..

[21] Micalet A., Tappouni L.J., Peszko K., Karagianni D., Lam A., Counsell J.R., Quezada S.A., Moeendarbary E., Cheema U. (2023). Urokinase-type plasminogen activator (uPA) regulates invasion and matrix remodelling in colorectal cancer. Matrix Biol. Plus.

[22] Heinemann V., Ebert M.P., Laubender R.P., Bevan P., Mala C., Boeck S. (2013). Phase II randomised proof-of-concept study of the urokinase inhibitor upamostat (WX-671) in combination with gemcitabine compared with gemcitabine alone in patients with non-resectable, locally advanced pancreatic cancer. Br. J. Cancer.

[23] Klijn J.G.M., Schmitt M., Magdolen V., Timmermans M., Sieuwerts A.M., Muehlenweg B., Schmalix W.A., Stürzebecher J., Setyono-Han B., Foekens J.A. (2017). Suppression of rat breast cancer metastasis and reduction of primary tumour growth by the small synthetic urokinase inhibitor WX-UK1. Thromb. Haemost..

[24] Wang D., Yang Y.S., Jiang L.G., Wang Y., Li J.Y., Andreasen P.A., Chen Z., Huang M.D., Xu P. (2019). Suppression of Tumor Growth and Metastases by Targeted Intervention in Urokinase Activity with Cyclic Peptides. J. Med. Chem..

[25] Xu P., Huang M.D. (2020). Small Peptides as Modulators of Serine Proteases. Curr. Med. Chem..

[26] Diao L., Meibohm B. (2013). Pharmacokinetics and Pharmacokinetic–Pharmacodynamic Correlations of Therapeutic Peptides. Clin. Pharmacokinet..

[27] Boruah A., Roy A. (2022). Advances in hybrid peptide-based self-assembly systems and their applications. Biomater. Sci..

[28] Wang X.J., Cheng J., Zhang L.Y., Zhang J.G. (2022). Self-assembling peptides-based nano-cargos for targeted chemotherapy and immunotherapy of tumors: Recent developments, challenges, and future perspectives. Drug Deliv..

[29] Singh D. (2025). Self-assembling Peptides Based Fibers: A Pioneering Innovation for Regenerative Medicine. J. Macromol. Sci. Part B-Phys..

[30] Yan S.C., Tang D., Hong Z.Y., Wang J., Yao H., Lu L., Yi H.M., Fu S.J., Zheng C.J., He G.C. (2021). CD133 peptide-conjugated pyropheophorbide-a as a novel photosensitizer for targeted photodynamic therapy in colorectal cancer stem cells. Biomater. Sci..

[31] Story S.C., Aldrich J.V. (2009). Preparation of protected peptide amides using the Fmoc chemical protocol. Int. J. Pept. Protein Res..

[32] Liu T.P., Wang B., Han Q., Gong W.S., Ye J. (2022). Dexmedetomidine protects gastric mucosal epithelial cells against ischemia/reperfusion-induced apoptosis by inhibiting HMGB1-mediated inflammation and oxidative stress. Trop. J. Pharm. Res..

[33] Li J., Cheng Y., Yang Y., Wang B., Jiang Y., Chen J., Li P., Du B. (2024). Toxic Effects of Arecoline on Human Stomach Mucosal Epithelial Cells. Sci. Technol. Food Ind..

[34] Brown K., Sugrue A., Modi K., Light R., Conley K., Cox A., Bender C., Miles S., Valentovic M., Dasgupta P. (2024). An Experimental Protocol for the Boyden Chamber Invasion Assay With Absorbance Readout. Bio-Protocol.

[35] Li W.P., Yen C.J., Wu B.S., Wong T.W. (2021). Recent Advances in Photodynamic Therapy for Deep-Seated Tumors with the Aid of Nanomedicine. Biomedicines.

[36] Tang L.L., Han X.Z. (2013). The urokinase plasminogen activator system in breast cancer invasion and metastasis. Biomed. Pharmacother..

[37] Dong F.D., Huang M.L., Li W.X., Liu T., Li L.X., Zuo S.Y., Zhang J.X., Xing J.L., Cui J.K., He Z.G. (2025). A dual-mode recognition strategy to enhance the lysosome-targeted bursting of PPa for efficient photodynamic cancer therapy. Nano Res..

[38] Hu Y.X., Peng J.F., Liu R., Gao J., Hua G.C., Fan X.J., Wang S.J. (2024). Porphyrin-Based Supramolecular Self-Assemblies: Construction, Charge Separation and Transfer, Stability, and Application in Photocatalysis. Molecules.

[39] George R.A., Heringa J. (2002). An analysis of protein domain linkers: Their classification and role in protein folding. Protein Eng..

[40] Li D., Ren J., Ji F., Peng Q., Teng H., Jia L. (2020). Peptide linker affecting the activity retention rate of VHH in immunosorbents. Biomolecules.

[41] Siwawannapong K., Zhang R., Lei H.L., Jin Q.T., Tang W.T., Dong Z.L., Lai R.Y., Liu Z., Kamkaew A., Cheng L. (2020). Ultra-small Pyropheophorbide-a Nanodots for Near-infrared Fluorescence/Photoacoustic Imaging-guided Photodynamic Therapy. Theranostics.

[42] Guidolin K., Ding L.L., Chen J., Wilson B.C., Zheng G. (2021). Porphyrin-lipid nanovesicles (Porphysomes) are effective photosensitizers for photodynamic therapy. Nanophotonics.

