# Supplementary material for: Dual-Functional Self-Assembled Nanoparticles for Synergistic Photodynamic Therapy and Antimetastatic Treatment of Colorectal Cancer
Source: Pharmaceutics. 2026 Jul 31;18(8):948. doi: 10.3390/pharmaceutics18080948 (PMC13516324; doi:10.3390/pharmaceutics18080948)
Supplement: Supplementary file 1 [file pharmaceutics-18-00948-s001.zip › pharmaceutics-4376926-supplementary.pdf]

Supporting Information

Dual-Functional Self-Assembled Nanoparticles for Synergistic Photodynamic Therapy and Antimetastatic Treatment of Colorectal Cancer

Table of Contents

Figure S1. Chemical structure of the PPA-IG2 conjugate.
Figure S2: HPLC chromatography of PPA-IG2.
Figure S3: Mass spectrum of PPA-IG2.
Figure S4: Concentration-dependent UV-Vis absorption spectra of PINPs.
Figure S5. Time-dependent cellular uptake of PINPs in normal GES-1 cells and tumor CT26 cells.
Figure S6. Ex vivo fluorescence imaging of tumors and major organs at different time points post-injection.
Figure S7. Photographs of excised tumors and body weight changes of mice in different treatment groups.

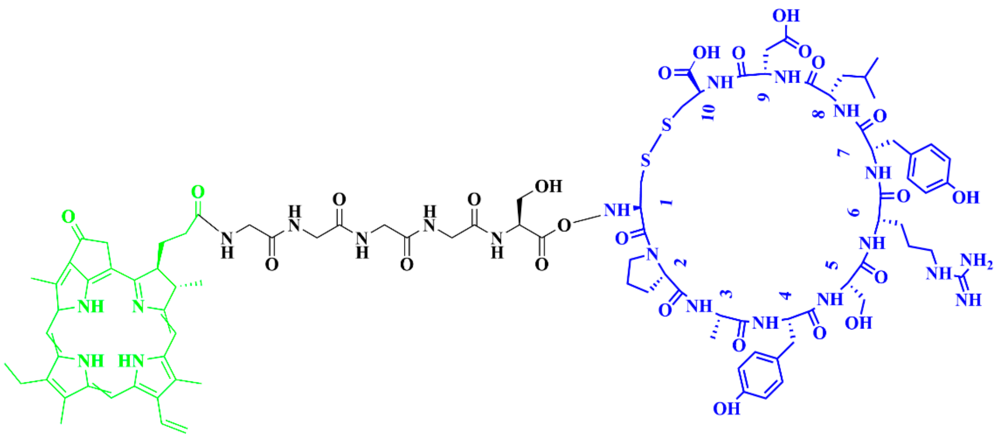

PPA   GGGGS-CPAYSRYLDC

Figure S1. Chemical structure of the PPA-IG2 conjugate. The chemical structure of PPA-IG2 conjugate synthesized by covalently linking pyropheophorbide-a (PPA) with the uPA-targeted cyclic peptide IG2.

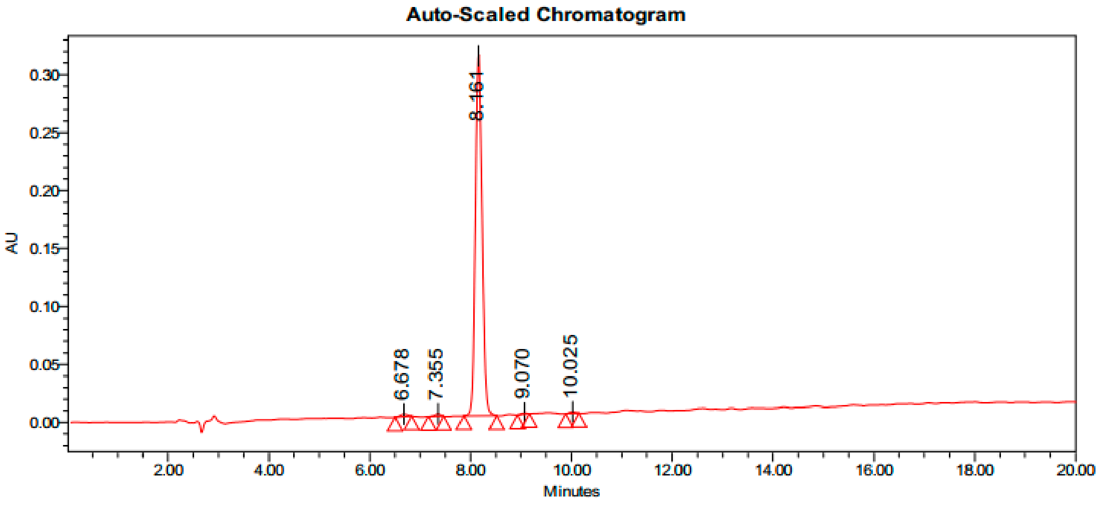

Processed Channel: 2487Channel 1

	Processed Channel	Retention Time (min)	Area	% Area	Height
1	2487Channel 1	6.678	21908	0.74	2145
2	2487Channel 1	7.355	16859	0.57	2103
3	2487Channel 1	8.161	2919584	98.06	312169
4	2487Channel 1	9.070	7638	0.26	1033
5	2487Channel 1	10.025	11358	0.38	1222

Figure S2: HPLC chromatography of PPA-IG2.

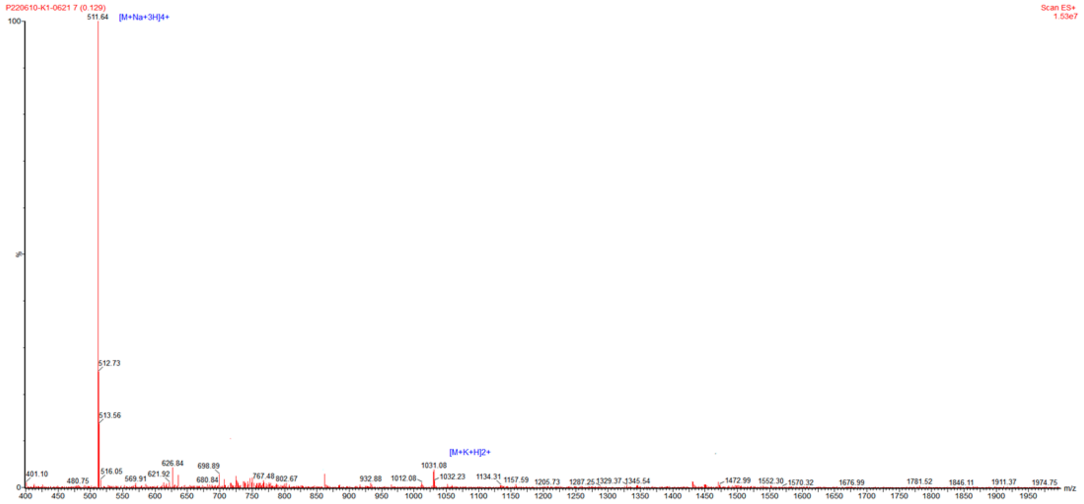

Sample Description		Instrument	Waters ZQ2000		
Analyzed date:	2022-08-19	Probe:	ESI	Probe Bias:	+4.5kv
Analyst:	PY	Nebulizer Gas Flow:	1.5L/min	Detector:	1.5kv
Sample:	焦脱镁叶绿酸-a-GGGGS-CPAYSRYIGC(二硫键)	CDL:	-20.0v	T. Flow:	0.2ml/min
M.W.:	2020.00	CDL Temp.:	250 °C	B. Conc.:	50%H2O/50%ACN
Lot. No.:	P220610-K1	Block Temp.:	200 °C		

Figure S3. Mass spectrum of PPA-IG2.

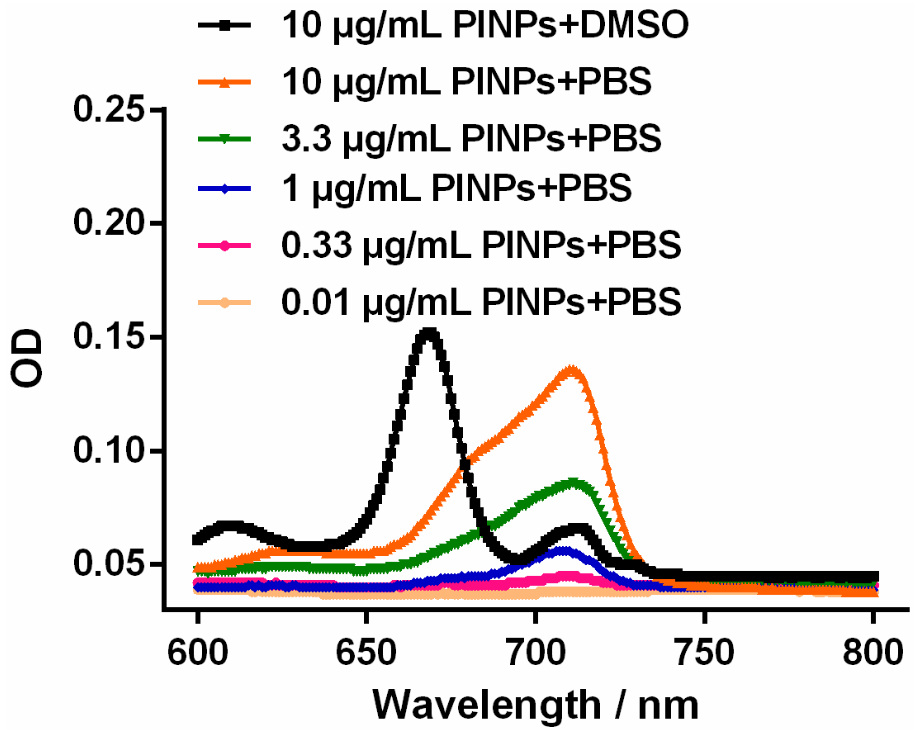

Figure S4. Concentration-dependent UV-Vis absorption spectra of PINPs. 10 μg/mL PINPs in DMSO served as the monomer control. Absorption spectra of PINPs in PBS at concentrations of 10, 3.3, 1, 0.33, and 0.01 μg/mL were measured to evaluate the concentration-dependent self-assembly behavior. The remarkable red shift of the main absorption peak in PBS relative to DMSO verifies the formation of π–π stacking-mediated J-type aggregates among PPA moieties.

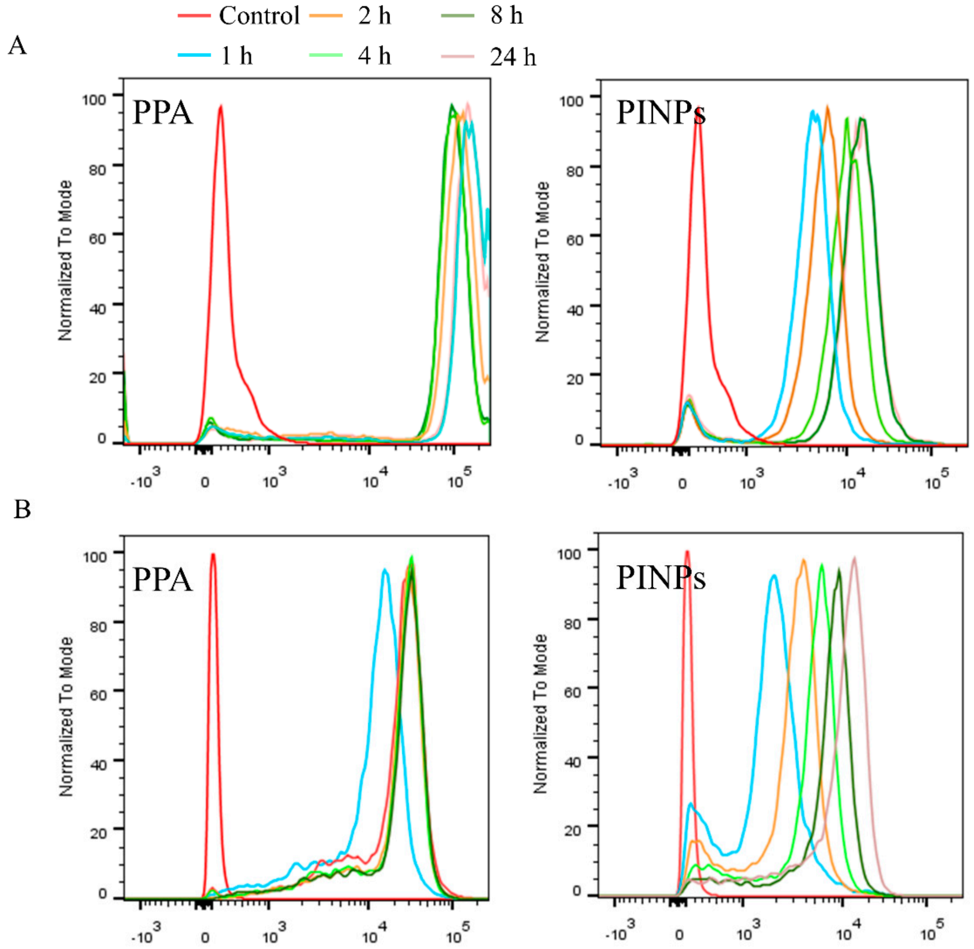

Figure S5. Time-dependent cellular uptake of PINPs in normal GES-1 cells and tumor CT26 cells. Flow cytometry analysis showing the time-dependent internalization of PINPs in human normal gastric mucosal GES-1 cells (A) and mouse colorectal cancer CT26 cells (B).

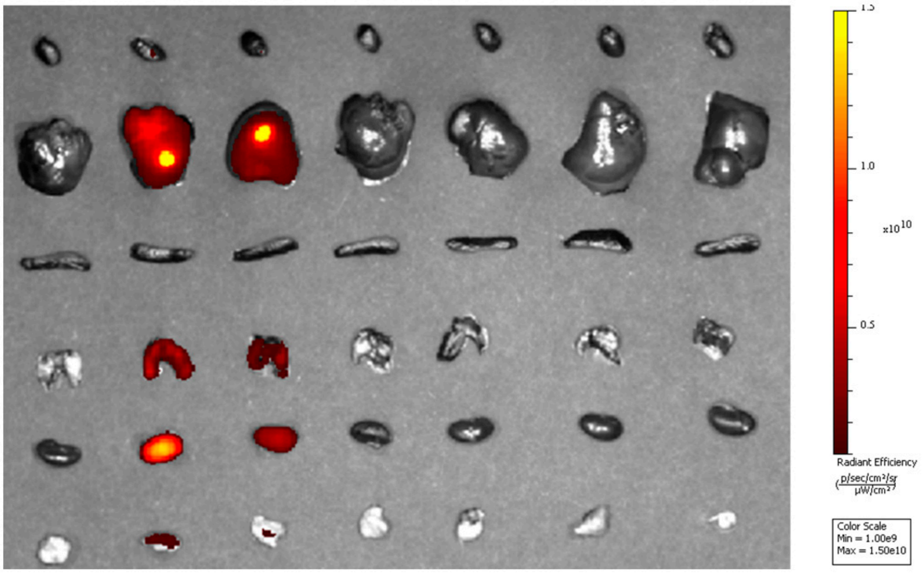

Figure S6. Ex vivo fluorescence imaging of tumors and major organs at different time points post-injection. Ex vivo fluorescence images of tumors, heart, liver, spleen, lung, and kidneys harvested from tumor-bearing mice at 0.5, 3, 6, 24, and 48 h after intravenous injection of PINPs.

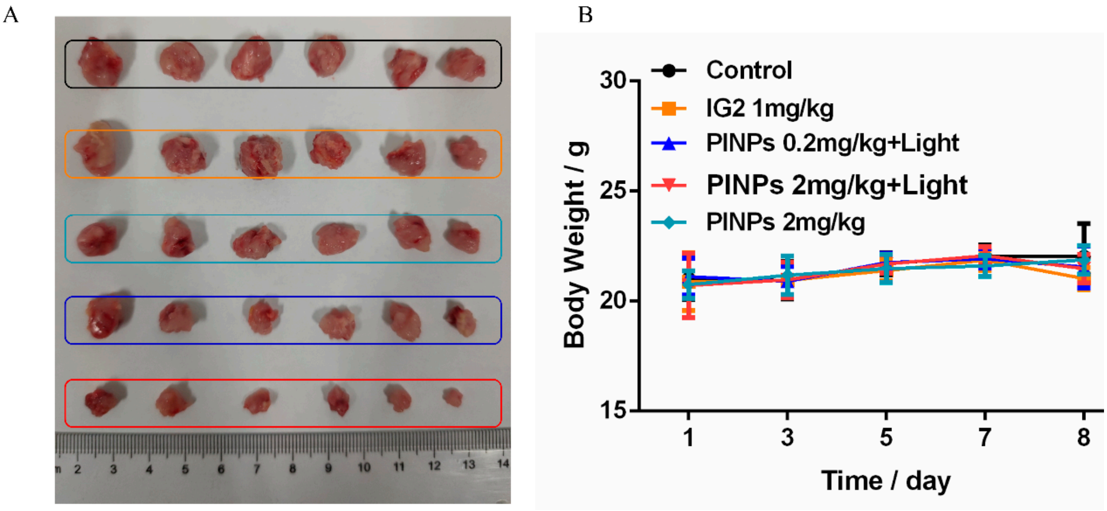

Figure S7. Photographs of excised tumors and body weight changes of mice in different treatment groups. (A) Representative digital photographs of tumors dissected from mice in each treatment group at the end of the therapy. (B) Body weight curves of mice recorded every day during the treatment period. Data are presented as mean ± SD (n = 6).
